# Posterior reversible encephalopathy syndrome complicated with subarachnoid hemorrhage in an eclamptic pregnant patient: case report

**DOI:** 10.1186/s12883-018-1186-1

**Published:** 2018-11-03

**Authors:** Dan Hu, Jing Xiong, Yunfei Zha, Zhaohui Zhang

**Affiliations:** 1 Department of Neurology, Renmin Hospital of Wuhan University, Jiefang Road 238, Wuchang District, Wuhan, 430060 China; 2 Department of Radiology, Renmin Hospital of Wuhan University, Jiefang Road 238, Wuchang District, Wuhan, 430060 China

**Keywords:** Posterior reversible encephalopathy syndrome, Subarachnoid hemorrhage, Eclampsia

## Abstract

**Background:**

Posterior reversible encephalopathy syndrome (PRES) is a neurotoxic condition which comprises various neurological symptoms. This syndrome could be complicated by intracranial hemorrhage including subarachnoid hemorrhage (SAH). However, SAH is rarely seen in eclamptic patients with PRES.

**Case presentation:**

A 34-weeks-pregnant woman at the age of 33 was admitted to the obstetrics department because of an episode of generalized tonic-clonic seizure. Before the seizure, the patient had a headache and was found to have an abnormal systolic blood pressure of 160 mmHg. On admission, systolic and diastolic blood pressures were up to 182 and 99 mmHg, respectively. Emergent cesarean section was then performed. On hospital day (HD) 2, cranial non-contrast computed tomography (CT) revealed the existence of SAH. Multiple areas of high signals on T2-weighted and fluid attenuated inversion recovery (FLAIR) sequences were shown by cranial magnetic resonance imaging (MRI) performed 2 days later. CT-angiography studies didn’t reveal intracranial aneurysm. After anti-hypertensive treatment, arterial blood pressure of the patient was gradually tapered to normal values. Eventually, the patient was discharged without any residual symptoms.

**Conclusions:**

SAH is a rare complication of PRES in eclamptic patients. In patients with PRES, occurrence of SAH is related to increased morbidity and mortality especially when the hemorrhage is diffuse or massive. Our patient had a minor hemorrhage. The good prognosis might also be due to immediate elimination of the risk factor of PRES by emergent delivery.

## Background

Posterior reversible encephalopathy syndrome (PRES) is a distinct clinico-radiological disease entity typically comprising a variety of symptoms such as headache, visual loss, impaired consciousness and epileptic seizures. This syndrome mainly occurs in the setting of hypertension, sepsis, eclampsia, autoimmune diseases or immunosuppressive therapy [1–3]. Cerebral imaging often present as a unique pattern of subcortical white matter edema with typical parietal-occipital predominance [4]. Recognition of subarachnoid hemorrhage as an atypical imaging appearance has recently increased [5, 6]. There are, however, few reports of such an occurrence in eclamptic patients with PRES. Here, we report a case of 34-weeks-pregnant woman who developed eclampsia and PRES as well as subarachnoid hemorrhage.

## Case presentation

**Fig. 1:**
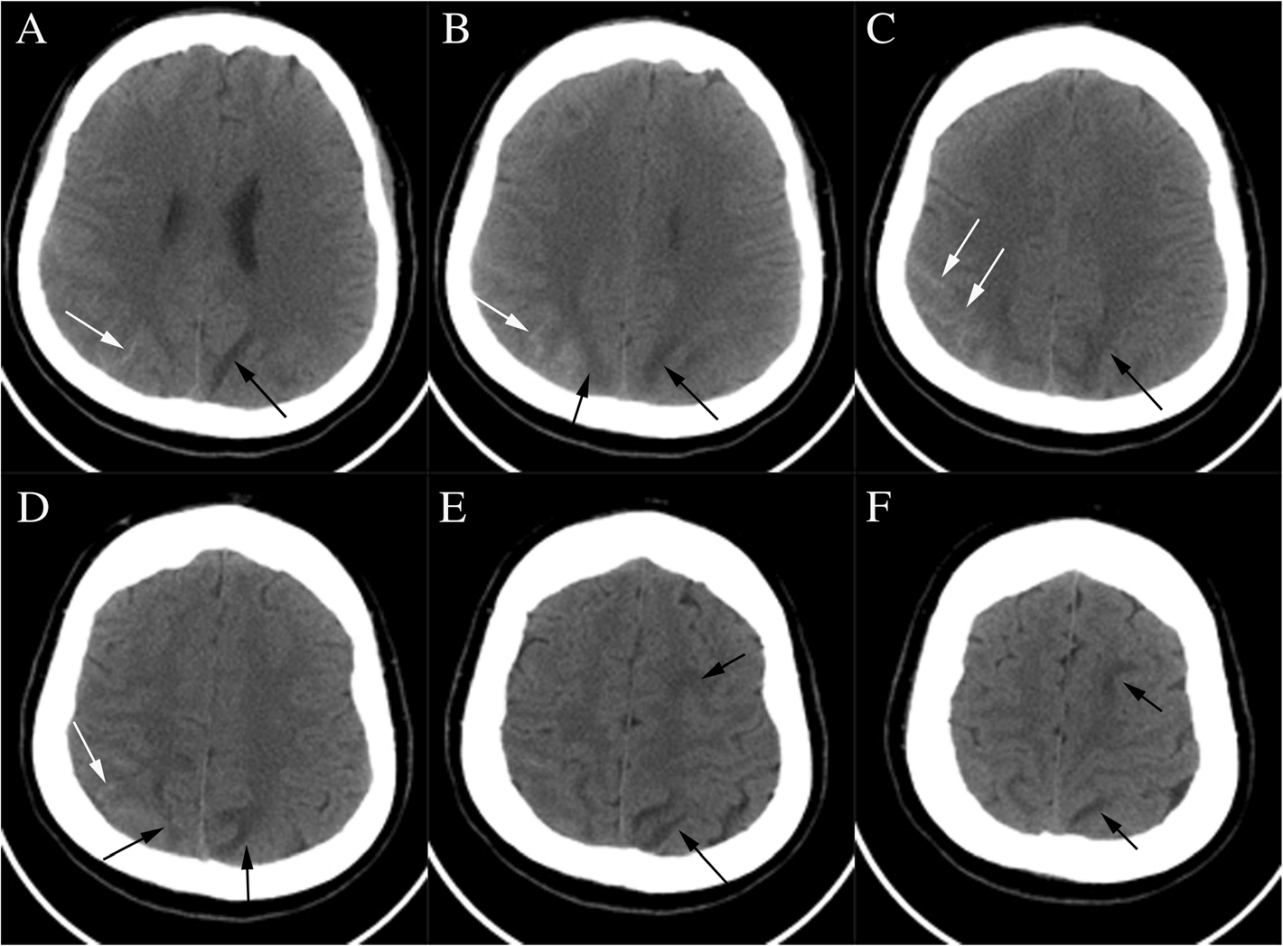
CT scan (**a-f**) shows lesions with hyperdensity in the parietal-occipital sulci (white arrows) and hypodensity in the left frontal and parietal lobes (black arrows)

**Fig. 2:**
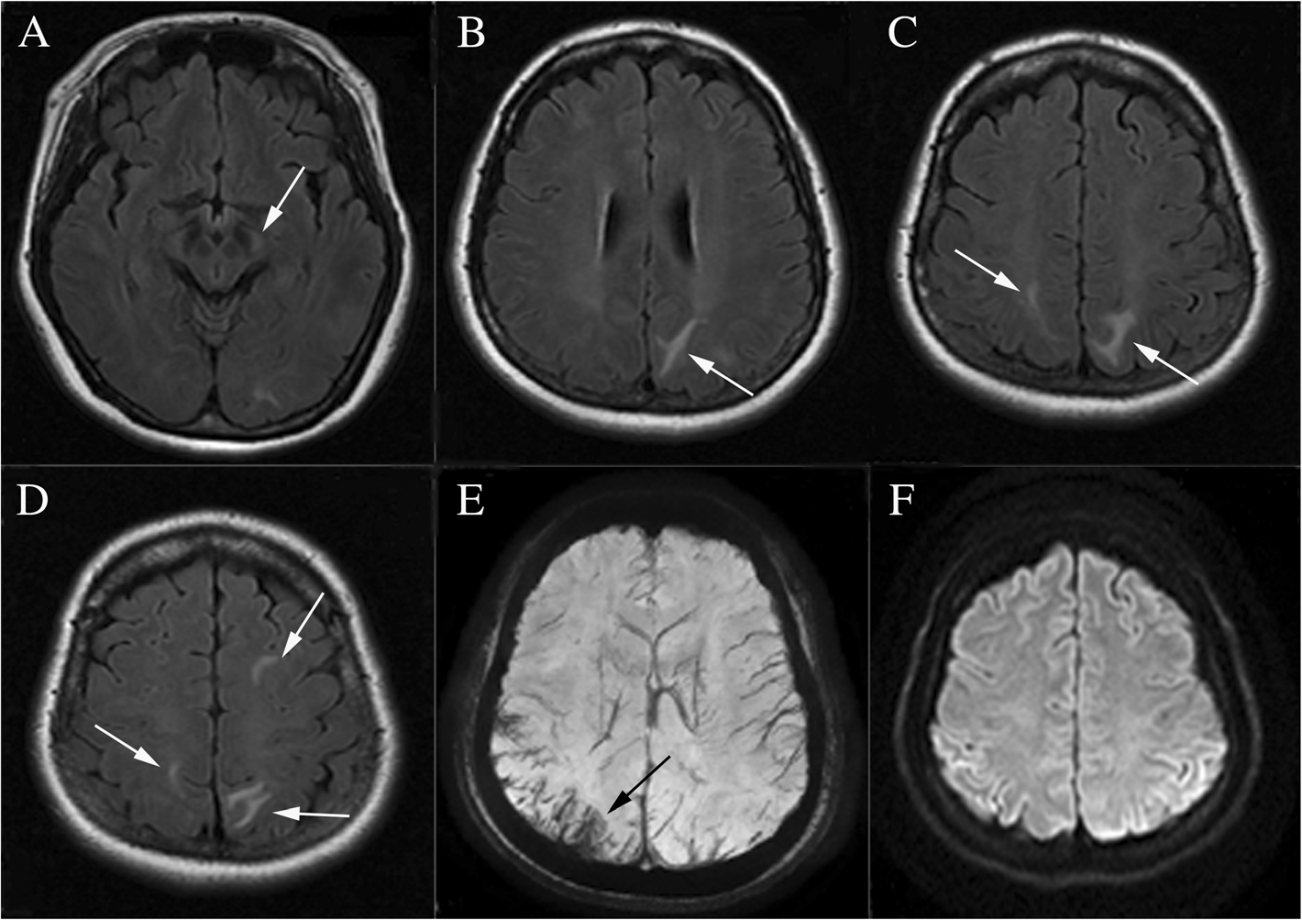
Axial FLAIR (**a**-**d**) demonstrates PRES lesions involving multiple regions (white arrows). SWI (**e**) reveals low signals in the right parietal-occipital sulci. There are no abnormal signals in DWI (**f**)

**Fig. 3:**
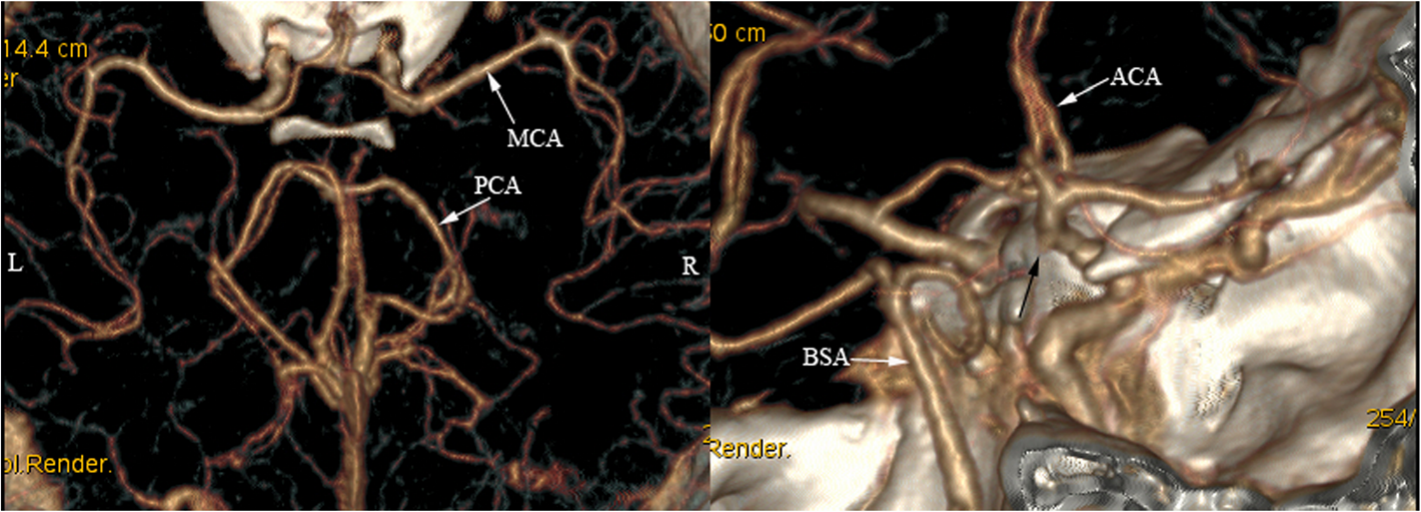
CTA shows a subtle enlargement at the origin of the right posterior communicating artery (black arrow). L: Left; R: Right; ACA: Anterior cerebral artery; MCA: Middle cerebral artery; PCA: Posterior cerebral artery; BSA: Basilar artery

A 34-weeks-pregnant woman at the age of 33 was admitted to the obstetrics department after having an episode of generalized tonic-clonic seizure accompanied by headache and transient blurred vision. 2 days before the occurrence of seizure, the patient had a headache which was relieved significantly after rest. Abnormal systolic blood pressure of 160 mmHg was discovered in the latest antenatal appointment. On admission, systolic and diastolic blood pressures were 182 and 99 mmHg, respectively [mean arterial pressure (MAP) 127]. On neurologic examination the patient was conscious. The laboratory studies disclosed leukocyte 12.9 × 10^9^/L (reference 3.5–9.5 × 10^9^/L) and lactate dehydrogenase (LDH) 282 U/L (reference 114–240 U/L). Clotting tests showed elevated levels of plasma D-dimer 3.61 mg/L (reference 0–0.55 mg/L) and fibrinogen 4.21 g/L (reference 2–4 g/L). The platelet count, the red blood cell count and levels of serum liver enzymes were all normal. Then, the patient was transferred to the operating room for an emergent cesarean section. The baby’s APGAR scores at 1 min, 5 min, and 10 min were 7, 8, and 8, respectively. On hospital day (HD) 2, the patient complained of abdominal pain without any other discomforts. Cranial non-contrast computed tomography (CT) revealed subarachnoid hemorrhage (SAH) in the right parietal-occipital sulci and hypodensities in the left frontal and parietal lobes (Fig. 1).Cranial magnetic resonance imaging (MRI) performed 2 days later showed multiple areas of high signals on T2-weighted and fluid attenuated inversion recovery (FLAIR) sequences, involving the left cerebral peduncle, the bilateral parietal and the left frontal subcortical regions (Fig. 2). There were no abnormal signals in the corresponding regions on diffusion weighted imaging (DWI) (Fig. 2). MRI susceptibility weighted imaging (SWI) confirmed the existence of SAH (Fig. 2). CT-angiography test performed thereafter was normal except for a subtle enlargement at the origin of the right posterior communicating artery (Fig. 3). During the hospitalization, the patient received oral anti-hypertensive drugs (Adalat, 30 mg per day) and arterial blood pressure was gradually tapered to normal range. When she was transferred to the department of neurology, no symptoms were observed and the neurologic examination was normal. About a few days later, a follow-up brain MRI (data not shown) showed no abnormality. Without use of any anti-hypertensive and anti-epileptic treatment, the patient was discharged eventually with normal blood pressure.

## Discussion and conclusions

PRES was first described by Hinchey and colleagues in 1996 [3]. Clinically, this disease is characterized by acute or subacute onset of neurological symptoms including altered mental state, epileptic seizures, headache, visual disturbances and focal neurological deficit (eg, hemiparesis, aphasia, and even myelopathic symptoms) [7]. Our patient presented with the constellation of symptoms including headache, blurred vision, and seizure. It has been proposed that generalized seizure is related to the focal abnormality of parietal-occipital lobes [2]. For our patient, MRI showed abnormal signals in the bilateral parietal subcortical regions, which might be responsible for occurrence of seizure.

The pathophysiology of PRES remains controversial. Rapid development of hypertension exceeds the upper limit of cerebral blood flow autoregulation, which leads to impaired cerebral autoregulation and hyperperfusion. Subsequently, the blood-brain barrier (BBB) breaks down, followed by extravasation of fluid and plasma proteins into the brain parenchyma [8]. Furthermore, the posterior head region is particularly affected because of poor sympathetic innervation in the posterior fossa. Another hypothesized mechanism involves endothelial dysfunction and vasoconstriction occurring secondary to systemic toxicity in eclampsia and sepsis, which are the common precipitants of PRES. Release of cytokines such as interleukin (IL)-1 and tumor necrosis factor (TNF-α) causes endothelial cell activation and damage, resulting in vasoconstriction and hypoperfusion [8–10]. It has been demonstrated that PRES imaging appearance commonly follows a watershed distribution [11]. Interestingly, acute hypertension without exceeding the upper limit of cerebral blood flow autoregulation (140–150 mmHg) could also lead to endothelial dysfunction in certain circumstances [12]. At least in the setting of eclampsia, however, endothelial dysfunction might contribute more to the development of PRES than hypertension, since there was no significant difference of the average MAP between the eclamptic patients with PRES and those without [13].

Radiologically, PRES typically presents as focal vasogenic edema and the posterior parietal-occipital white matter is commonly affected. Frequent frontal and temporal lobe involvement has also been reported [14]. Atypical manifestations include the location of lesions in the cerebellum, basal ganglia and brainstem [15]. MRI of our patient showed abnormal T2 and FLAIR signals in the left cerebral peduncle except for the typical locations. In patients with eclampsia, the involvement of atypical brain regions might be explained by the relatively significant impact of cytokine mediated endothelial dysfunction with respect of hypertension on the development of BBB breakdown [13].

Intracranial hemorrhage is common in PRES, which has been reported with an incidence ranging from 9 to 33% [16–18]. As for the types of intracranial hemorrhage, SAH is relatively less common than intraparenchymal hemorrhage. Notably, the blood amount of SAH in PRES is mostly minimal or moderate and basal cisterns are usually spared, which is distinct from that in cases of aneurysmal rupture [5]. PRES-related hemorrhage is usually to be observed in patients with eclampsia, sepsis/recent infection, allogeneic bone marrow transplantation and those undergoing therapeutic anticoagulation [5, 12, 19]. By contrast, hypertension does not appear to be a prerequisite for the occurrence of hemorrhage [12]. The mechanism behind hemorrhage in PRES is not fully elucidated. It has been proposed that hemodynamic disturbances, endothelial dysfunction, blood brain barrier breakdown and abnormal coagulation profile might play roles in the development of hemorrhage in the setting of PRES [5]. Voetsch et al. [20] reported a case of PRES which is caused by SAH-associated vasospasm. In that case, the blood of SAH presented as a thick clot in the right Sylvian fissure and cerebral angiogram revealed a wide-necked 3 mm right middle cerebral artery (MCA) aneurysm. Aneurysmal SAH-induced PRES was also reported in another literature [21]. All of the reported patients manifested as diffuse acute SAH in basal cisterns resulting from a ruptured anterior communicating artery aneurysm. In contrast, our patient only showed a minor SAH at the cerebral convexities and subsequent CT-angiography studies did not show intracranial aneurysm. Thus, SAH was more likely to be a complication rather than a cause of PRES in our case. Reversible cerebral vasoconstriction syndrome (RCVS) is clinically characterized by thunderclap headaches. Patients with RCVS could also present with seizures and focal neurological deficits similar to that in PRES. Some of the cases are even accompanied by SAH and PRES [22]. However, diffuse segmental vasoconstrictions of the cerebral arteries, the radiological characteristic of RCVS, were not found on CT-angiography in our case.

Additionally, parenchymal edema was usually located in the ipsilateral cerebral lobe with respect to SAH in the eclamptic patients with PRES [5]. In our case, however, parenchymal edema predominantly occurred in the cerebral lobe contralateral to SAH, which is rarely seen in these cases. To our knowledge, only one reported eclamptic patient developing PRES presented with this discordant location rediologically [5].

Generally speaking, PRES has a favorable prognosis and the disorder is reversible when the predisposing factors are treated. However, it has been identified that the clinical outcome in patients with PRES is related to multiple factors including intracranial hemorrhage [23]. Hemorrhage appears to lead to increased morbidity and mortality, while it is difficult to determine the relationship in cases of minor hemorrhage [19, 23]. Our patient had a complete resolution of symptoms with a small amount of SAH, which might be associated with emergent delivery.

In the present paper, we described a case of an eclamptic patient who was diagnosed with PRES and SAH. It is rare for PRES-related SAH to occur in pregnant patients with eclampsia. This case provides us with information regarding the diagnosis, treatment, and prognosis of this kind of patients.

## Abbreviations

BBB: Blood-brain barrier
CT: Computed tomography
DWI: Diffusion weighted imaging
FLAIR: Fluid attenuated inversion recovery
LDH: Lactate dehydrogenase
MAP: Mean arterial pressure
MRI: magnetic resonance imaging
PRES: Posterior reversible encephalopathy syndrome
RCVS: Reversible cerebral vasoconstriction syndrome
SAH: Subarachnoid hemorrhage
SWI: Susceptibility weighted imaging
